# Evaluation of Cytotoxic and Antimicrobial Effects of Two *Bt* Cry Proteins on a GMO Safety Perspective

**DOI:** 10.1155/2014/810490

**Published:** 2014-07-23

**Authors:** Davi Felipe Farias, Martônio Ponte Viana, Gustavo Ramos de Oliveira, Magda Aparecida Beneventi, Bruno Marques Soares, Claudia Pessoa, Igor Parra Pessoa, Luciano Paulino Silva, Ilka Maria Vasconcelos, Maria Fátima Grossi de Sá, Ana Fontenele Urano Carvalho

**Affiliations:** ^1^Graduate Program in Biochemistry, Federal University of Ceará, 60440-900 Fortaleza, CE, Brazil; ^2^Graduate Program in Bioprocess Engineering and Biotechnology, Federal University of Paraná, P.O. Box 19011, 81531-98 Curitiba, PR, Brazil; ^3^National Center of Genetic Resources (Embrapa-Cenargen), Parque Estação Biológica-PqEB-Avenida, W5 Norte (Final), P.O. Box 02372, 70770-917 Brasília, DF, Brazil; ^4^Graduate Program in Pharmacology, Federal University of Ceará, 60430-270 Fortaleza, CE, Brazil; ^5^Graduate Program in Gemonics Sciences and Biotechnology, Catholic University of Brasília, SGAN Quadra 916, Módulo B, W5 Norte, Asa Norte, 70790-160 Brasília, DF, Brazil

## Abstract

Studies have contested the innocuousness of *Bacillus thuringiensis (Bt)* Cry proteins to mammalian cells as well as to mammals microbiota. Thus, this study aimed to evaluate the cytotoxic and antimicrobial effects of two Cry proteins, Cry8Ka5 (a novel mutant protein) and Cry1Ac (a widely distributed protein in GM crops). Evaluation of cyto- and genotoxicity in human lymphocytes was performed as well as hemolytic activity coupled with cellular membrane topography analysis in mammal erythrocytes. Effects of Cry8Ka5 and Cry1Ac upon *Artemia* sp. nauplii and upon bacteria and yeast growth were assessed. The toxins caused no significant effects on the viability (IC_50_ > 1,000 *µ*g/mL) or to the cellular DNA integrity of lymphocytes (no effects at 1,000 *µ*g/mL). The Cry8Ka5 and Cry1Ac proteins did not cause severe damage to erythrocytes, neither with hemolysis (IC_50_ > 1,000 *µ*g/mL) nor with alterations in the membrane. Likewise, the Cry8Ka5 and Cry1Ac proteins presented high LC_50_ (755.11 and >1,000 *µ*g/mL, resp.) on the brine shrimp lethality assay and showed no growth inhibition of the microorganisms tested (MIC > 1,000 *µ*g/mL). This study contributed with valuable information on the effects of Cry8Ka5 and Cry1Ac proteins on nontarget organisms, which reinforce their potential for safe biotechnological applications.

## 1. Introduction

In the latest years, advances in genetic engineering led to the development of plants that present resistance to insects through expression of* cry* genes from* Bacillus thuringiensis *(*Bt*), a soil Gram-positive bacteria. Various plant species, particularly those of economic relevance, such as corn, cotton, potato, tobacco, tomato, and sugar cane, have been genetically modified to express Cry proteins [1–3]. Among these proteins, Cry1Ac was one of the first to be used for the development of* Bt *cultures. Many of these Cry1Ac* Bt *crops are still available in the biotech seed market [2, 4, 5].

Recently, it has been engineered the Cry8Ka5 mutant protein which is active against the cotton boll weevil (*A. grandis*), the main pest of Brazilian cotton culture. This new protein was obtained by* in vitro* directed molecular evolution technology, DNA shuffling, applied to the* cry8Ka*1 gene cloned from the Brazilian* Bt* strain S811 [6]. Due to its promising activity, Cry8Ka5 is a potential candidate to develop a cotton resistant to* A. grandis *attack. However, biopesticides, including recombinant proteins, must be rigorously tested for their safety for humans and animals [7] and their effects upon nontarget organisms must be thoroughly verified [8, 9].

It has been widely reported by scientific literature that* Bt* Cry toxins do not cause adverse effects to mammalian cells, since they do not possess specific receptors for Cry proteins binding [10–12]. On the other hand, studies have contested the innocuous nature of these proteins and some equivalent receptors have also been described to mammalian cells [13, 14]. Furthermore, it is widely known the antimicrobial property of* Bt* Cry toxins, which raises a concern about the effects of these proteins upon the microbiota of mammals gastrointestinal tract, whether monogastric or ruminants [15–17]. Therefore, given the extensive development and increasing use of GM cultures, including edible plant species expressing* Bt* Cry proteins, new complementary or alternative viable methods, especially those in vitro, for safety assessment of GM foods [18, 19].

In order to contribute with safety additional information of Cry1Ac and Cry8Ka5 toxins on nontarget organisms, this study aimed to evaluate those two entomotoxins for the presence of cytotoxic and antimicrobial effects. Among the cytotoxicity tests, evaluation of cytotoxicity and genotoxicity in human peripheral lymphocytes and hemolytic activity assay of mouse, rabbit, and human (types A, B, AB, and O) erythrocytes, coupled with cellular membrane topography analysis performed by atomic force microscopy (AFM), have been run. As an alternative and broadly used test in screenings for cytotoxic compounds, the effects of Cry8Ka5 and Cry1Ac on the survival of* Artemia* sp. nauplii were also assessed. Finally, we evaluated the effects of these proteins on bacteria and yeast growth in liquid medium.

## 2. Materials and Methods

### 2.1. Protein Production and Characterization

The Cry8Ka5 protein (approximate molecular mass of 73 kDa) was obtained through the expression of the* cry8Ka5* gene in* E. coli* BL21 (DE3) containing the mutant gene inserted into PET101/D TOPO plasmid (Invitrogen) as described by Oliveira et al. [6]. The Cry1Ac protein was firstly obtained as a protoxin (approximate molecular mass of 130 kDa) through heterologous expression in* E. coli *JM109 (Promega) transformed with the recombinant plasmid pKK (Amershan) containing the* cry1Ac* gene of the* Bt* var.* kurstaki* HD73 (data not published). Cells were grown in liquid LB medium containing ampicillin (1 *μ*g/mL) at 37°C, under continuous stirring at 175 rpm. To induce expression, 1 mM IPTG was applied to the culture when the OD_600_ reached 0.8. After 72 h of induction, cells were collected by centrifugation (4,500 × *g*, for 20 min, at 10°C) and the pellet was resuspended in lysis buffer (sucrose 15%, lysozyme 2 mg/mL, EDTA 50 mM, Tris HCl 50 mM, pH 8.0) for 12 h, at 4°C. The cell suspension was sonicated on ice (5 cycles of 300 s at 70%) and centrifuged for 10,000 × *g*, for 15 min, at 4°C. The pellet was then washed by centrifugation (10,000 × *g*, for 30 min, at 4°C) three times in 30 mL of 0.5 M NaCl solution containing 2% Triton X-100, five times in 30 mL of NaCl 0.5 M and twice with 30 mL of distilled water. Then, the inclusion bodies containing the Cry1Ac protoxin were solubilized in solubilization buffer (10 mM DTT, 50 mM sodium carbonate, and pH 10.5) for 2 h, at 37°C, and dialyzed against water in a 12 kDa dialysis membrane. The protoxin was activated with trypsin 1 : 50  (w/w) for 18 h, at 37°C. The proteolysis was stopped with 1 mM PMSF. The material containing the active toxin Cry1Ac (approximate molecular mass of 65 kDa) was again dialyzed with 12 kDa dialysis membrane against distilled water for 24 h.

The production of both proteins was monitored through quantification of soluble proteins by the Bradford method [20], using a curve constructed with bovine serum albumin as standard. Besides, the purity of Cry8Ka5 and Cry1Ac and activation of the last one were monitored by 12.5% SDS-PAGE [21].

From the SDS-PAGE gels of Cry8Ka5 and Cry1Ac the relative percentage of purity of both proteins was calculated using the Image Master 2D platinum (v.7.0, GE Healthcare) software. This methodology has been successfully adopted by our research team, and it is also widely used by chemical companies to show the purity of the commercialized proteins.

Before the use in the cytotoxic, genotoxic, and antimicrobial tests, both proteins were also checked for their bioactivity against their respective target insects. The Cry8Ka5 protein was tested against* A. grandis* larvae according to the methodology described by Oliveira et al. [6] and the Cry1Ac against* Spodoptera frugiperda* larvae following the method described by Grossi-de-Sa et al. [22].

### 2.2. N-Terminal Sequence Determination

To confirm the identity of the proteins obtained, the N-terminal amino acid sequences of the Cry8Ka5 and Cry1Ac proteins were determined on a Shimadzu PPSQ-10 automated protein sequencer (Kyoto, Japan) performing Edman degradation [23]. The sequences were determined from protein blotted on polyvinylidene fluoride (PVDF) after Tricine-SDS-PAGE. Phenylthiohydantoin (PTH) amino acids were detected at 269 nm after separation on a reverse phase C18 column (4.6 mm × 2.5 mm) under isocratic conditions, according to the manufacturer's instructions. The sequences obtained were compared with available amino acids sequences at UniProtKB database (http://www.uniprot.org/) using the FASTA3 search program. In addition, the sequences were then aligned in the Uniprot platform (http://www.uniprot.org/) to the complete sequences of Cry8Ka5 and Cry1Ac previously determined by our group.

### 2.3. Evaluation of Cyto- and Genotoxicity in Human Lymphocytes 

#### 2.3.1. Peripheral Blood Collection and Isolation of the Peripheral Blood Mononuclear Cells

Peripheral blood sampling was performed using sterile disposable 10 mL syringe or safety needle (BD vacutainer). To this end, three healthy adult volunteers were chosen, of either sex, aged 20–30 years, with no history of recent illness, no smoking, and no recent exposure to radiation, drug, or alcohol [24].

The peripheral blood mononuclear cells were isolated from a sample of 3 mL peripheral blood plus 5 mL of 50 mM sodium phosphate buffer, pH 7.5. This mixture was added to a graduated tube with 2 mL of Ficoll and subjected to centrifugation at 2,000 × *g*, for 30 min, at 25°C. Then, the intermediate region between the red cells and serum, called “lymphocytes cloud,” was aspirated and transferred to a third tube. Subsequently, it was filled with 50 mM sodium phosphate buffer, pH 7.5, to a final volume of 11 mL, and the tube was centrifuged at 108 × *g*, for 20 min, at 25°C. The supernatant was discarded and the pellet of lymphocytes was resuspended in 2 mL of 50 mM sodium phosphate buffer, pH 7.5.

#### 2.3.2. Cytotoxic Activity in Human Lymphocytes

The evaluation of the cytotoxic potential of the Cry8Ka5 and Cry1Ac proteins in human lymphocytes was determined by the MTT [3-(4,5-dimethylthiazol-2-yl)-2,5-diphenyltetrazolium bromide] method as described by Mosmann [25]. The lymphocytes obtained from healthy individuals were cultured in 96-well microplates at a density of 2 × 10^6^ cells/mL. After 24 h of the culture starting point, lymphocytes were exposed for 72 h to the proteins at concentrations ranging from 1.5 to 1,000 mg/mL. A 20 mM sodium phosphate buffer, pH 7.0, was used as negative control and doxorubicin (0.6 mM) was used as positive control. After the incubation period, the plates were centrifuged at 15 × *g*, for 15 min, at 25°C, and the supernatant was discarded. Each well received 200 *μ*L of MTT solution (10% in RPMI 1640) and was reincubated for 3 h, at 37°C, under controlled atmosphere of 5% CO_2_. After this period, the plates were again centrifuged (30 × *g*, for 10 min, at 25°C), the supernatant was discarded, and the precipitate was resuspended in 150 *μ*L of DMSO. To quantify the reduced salt in living cells, the absorbance was measured with the aid of a microplate spectrophotometer at a wavelength of 550 nm. IC_50_ values (inhibitory concentration of 50% of cells) and confidence interval (CI of 95%) were calculated by nonlinear regression analysis. Significant differences between means were calculated by analysis of variance (ANOVA) followed by Student-Newman-Keuls (*P* < 0.05), using appropriate statistical software.

#### 2.3.3. Genotoxicity Activity in Human Lymphocytes

The comet assay, also known as single cell gel electrophoresis (SCGE), has been routinely used in the study of the genotoxic potential of industrial chemicals, biocides, agrochemicals, and pharmaceuticals. According to the protocol described by Lima et al. [27], lymphocytes obtained from healthy individuals (5.0 × 10^4^ cells/mL) were cultured in the presence of increasing concentrations (100, 500, and 1,000 *μ*g/mL) of both proteins, Cry8Ka5 and Cry1Ac, for 24 h, at 37°C, in an atmosphere of 5% CO_2_. A 20 mM sodium phosphate buffer, pH 7.0, was used as negative control and doxorubicin (0.6 mM) as positive control. After treatment, cells were subjected to agarose gel electrophoresis (25 V, 300 mA) for 20 min. For staining the gel slides, a solution of ethidium bromide (20 mg/mL) was used. The slides were analyzed with the aid of a fluorescence microscope (Olympus, model BX41, Shinjuku, Tokyo, Japan). The analysis was performed according to the standard scores previously determined by the size and intensity of the comet tail. One hundred comets were counted and classified per slide by visual analysis. The results were organized in five categories (0, 1, 2, 3, and 4) that represent the percentage of DNA in the comet tail, which in turn indicates the degree of injury suffered by the cell. The damage index (DI) was calculated by the following formula:

(1) $$\begin{array}{c} \text{DI}=\overset{4}{\underset{i=0}{\sum }}n_{i}\times i, \end{array}$$

where *n*_*i*_ is the number of cells with the degree of injury *i* (0, 1, 2, 3, or 4).

Thus, DI values can range from 0 (no injury) to 4 (maximum injury). For statistical analysis, significant differences between means were calculated by analysis of variance (ANOVA) followed by Student-Newman-Keuls (*P* < 0.05), using suitable statistical software.

### 2.4. Hemolytic Activity in Erythrocytes of Humans and Laboratory Animals

#### 2.4.1. Human Donors

The human blood of A, B, AB, and O blood types was obtained from adult volunteer donors, three volunteers for each blood type of any gender, 20–30 years, in healthy conditions as described in Section 2.3.1.

All procedures involving humans were submitted for approval by the Ethics Committee on Human Research of the Federal University of Ceará (Fortaleza, Brazil) to be conducted in accordance with Resolution 196/96 of the National Health Council.

#### 2.4.2. Laboratory Animals

One male California rabbit with three months of age was purchased from the Cuniculture Division of the Federal University of Ceará (Fortaleza, Brazil). The rabbit was maintained in the Biochemistry and Molecular Biology Department, at the same Institution, with water and food (Biobase, São Paulo, Brazil)* ad libitum*.

Three female Wistar rats with three weeks of age were obtained from the Animal Facilities of the Federal University of Ceará (Fortaleza, Brazil). The animals were housed in the Department of Biology, at the same University, with temperature 23.0 ± 2.0°C, photoperiod (12 h dark/12 h light) and humidity (45–55%) controlled. The rats were kept in adequate numbers in boxes of polypropylene with substrate of pine wood shavings. Water and food were offered* ad libitum* until the approximate weight of 120 g for blood sampling is reached.

All protocols with animals adopted in this work were submitted for approval by the Ethics Committee on Animal Research of the Federal University of Ceará (Fortaleza, Brazil), that follows the principles of the Brazilian College of Animal Experimentation and obeys the Law N° 11.794 of 8 October 2008 (Lei Arouca), which regulates the use of animals in scientific research.

#### 2.4.3. Blood Sampling

The human blood was collected from the antecubital area of the arm using sterile disposable 10 mL syringe or safety needle, and transferred to heparinized tubes, slowly homogenized and stored at 4°C until the time of isolation of erythrocytes.

The rabbit blood was collected from a small cross-section in the marginal ear vein with a scalpel. The blood was collected in heparinized tube, homogenized, and conditioned at 4°C until the isolation of erythrocytes. To sample mice blood, the animals were slightly sedated with halothane (Zeneca, São Paulo, Brazil), and blood was collected by puncturing the retro-orbital plexus with a glass microhematocrit tubes. Blood was collected in heparinized microtubes, homogenized, and maintained at 4°C.

A portion of the human type O blood was set aside for cell membrane topography analysis, while the other part was intended for isolation of erythrocytes. All other blood samples were destined only to isolation of the erythrocytes. Then, for the isolation of erythrocytes and preparation of a suspension at 2%, all blood samples were centrifuged at 200 ×*g*, for 10 min, at 25°C. The supernatant was discarded and from the precipitate was removed 2 mL, which was mixed with 100 mL of 0.9% NaCl. The suspension was slightly mixed and packed at 4°C for no more than 24 h.

#### 2.4.4. Hemolytic Activity

The hemolytic activity of the entomotoxins was investigated following the methodology described by Merker and Levine [28] and Bernheimer [29], with some modifications. The presence of hemolysis in rat, rabbit, and human (types A, B, AB, and O) erythrocytes suspensions at 2% was observed after incubation for 1 h (30 min at 37°C and then 30 min at 25°C) with increasing concentrations of Cry8Ka5 and Cry1Ac proteins, ranging from 7.8 to 1,000 *μ*g/mL in a ratio of 1 : 2  (v/v). Bovine serum albumin was used as control in the same conditions. The degree of hemolysis was calculated by measuring the release of hemoglobin at a wavelength of 540 nm after centrifuging the mixture at 1,000 × *g*, for 5 min, at 25°C. The complete lysis (100%) was obtained by diluting 200 *μ*L of the 2% cell suspension in 200 *μ*L of distilled water, and no lysis (negative control) was obtained with the same proportion of cells in saline solution. Hemolytic activity was calculated according to the following formula:

(2) [(Abs540  of  the  sample)−(Abs540  of  the  negative  control)]  ×100Abs540  of  the  positive  control.

IC_50_ values (concentration causing 50% hemolysis of the cells) and confidence interval (CI of 95%) were calculated by analysis of nonlinear regression. Significant differences between means were calculated by analysis of variance (ANOVA) followed by Student-Newman-Keuls (*P* < 0.05), using proper statistical software.

### 2.5. Analysis of the Cellular Membrane Topography by AFM

To assess any effects of the Cry8Ka5 and Cry1Ac proteins on erythrocytes which do not necessarily trigger hemolysis, data from cellular membrane topography obtained by AFM were analyzed to provide additional information to the classical hemolytic activity assay. For that, it was used human whole blood O type treated with Cry8Ka5 or Cry1Ac (1,000 *μ*g/mL) for 30 min at 37°C and then for 30 min at 25°C. For comparison purposes, the cells were also treated with BSA (1,000 *μ*g/mL) or were just untreated. Then, we prepared blood films on slides fixed with methanol and allowed them to air dry, for all treatments (three slides per treatment).

The images were obtained in the constant contact force mode using cantilevers of 200 *μ*m in “V” shape (spring constant value of ≫0.15 N/m and resonance frequency of 24 kHz) and integrated pyramidal tip (radius of curvature <20 nm). The movement of the scanner was 100 *μ*m in *XY* directions and 7 *μ*m in the direction *Z*. All images were acquired as 512 × 512 pixels at a scanning rate of 1 Hz. The images were processed by the software Particle Analysis SPM-9600 (Shimadzu, Tokyo, Japan). The process consisted of an automatic levelling Wt surface. One hundred individual cells from each treatment were manually segmented using the function of markup analysis program, followed by the cell measurements. The parameters selected for the measurements were (1) perimeter, which obtains the contour length of a particle and the default width obtained by measuring the distance between two parallel lines which are tangent to a particle; (2) average *Z*, which obtains the average value data from the *Z* pixels that form a particle; (3) area excluding voids which provides an area using the equation: Area excluding voids = (area per pixel) × (number of pixels of a particle); (4) area including voids which provides an area using the equation: area including voids = (area per pixel) × [(number of pixels of a particle) + (number of pixels in the void of a particle)]; (5) surface area (the calculation uses imaginary structures where the points of the structure are created from *Z* values spatially arranged from pixels that form a particle, and quadrangles formed with the points of the structures are divided into two to create triangles; then, the sum of the areas of these triangles is calculated); and (6) volume which is obtained by the following equation: volume = area excluding voids × average of *Z* value.

#### 2.5.1. AFM Data Analysis

The parameters processed by the software Particle Analysis SPM-9600 (Shimadzu, Japan) were subjected to one-way ANOVA statistical analysis, and the means were compared by Sidak multiple comparisons (*P* < 0.05), using the appropriate statistical program.

### 2.6. Effects on* Artemia* sp

#### 2.6.1. *Artemia* Nauplii

Cysts of brine shrimp were purchased from aquarium shops of the Fortaleza city (Ceará, Brazil). To obtain brine shrimp nauplii, a small portion of cysts was placed to hatch in 2 L of artificial seawater (15.15 g NaCl + 2.98 g MgCl_2_ *·*6H_2_O + 2.86 g MgSO_4_ *·*7H_2_O + 0.65 g CaCl_2 _+ 0.116 g NaHCO_3_ + 0.414 g KCl, in 1 L of dechlorinated distilled water), under constant artificial illumination until 48 h before the beginning of the experiment. No food was administered during this period.

#### 2.6.2. Cytotoxicity Activity towards* Artemia* sp

The effects of Cry8Ka5 and Cry1Ac proteins at concentrations ranging from 3.9 to 1,000 *μ*g/mL on the survival rate of* Artemia* sp. were analyzed. The brine shrimp lethality assay is proposed as an alternative model for evaluation of cytotoxicity [30]. Brine shrimp nauplii (*n* = 10/sample/concentration) hatched in artificial seawater (salinity of 3.5%) and after 48 h they were transferred to a 3.0 mL volume of each sample at different concentrations. The samples were previously prepared in seawater. Negative controls were prepared using just seawater or seawater containing BSA at 1,000 *μ*g/mL.

After 24 h, the dead nauplii were counted at each concentration of the samples and plotted on a graph to correlate mortality × sample concentration, to calculate the lethal concentration for 50% of individuals (LC_50_) by Probit analysis [31].

### 2.7. Effects on Microorganisms

#### 2.7.1. Microorganisms

Two strains of Gram-positive bacteria,* Bacillus subtilis* ATCC 6633 and* Staphylococcus aureus* ATCC 25923, and two strains of Gram-negative,* Enterobacter aerogenes* ATCC 13048 and* Salmonella choleraesuis* ATCC 10708, were provided by the Laboratory of Microbial Ecology and Biotechnology from the Federal University of Ceará (Fortaleza, Brazil) and were initially purchased from ATCC (American Type Culture Collection, Manassas, VA, USA). The yeast* Candida albicans, C. tropicalis,* and* Pichia anomala* were obtained, identified, isolated, and maintained in the same Laboratory, while* Saccharomyces cerevisiae* was obtained commercially in the form of yeast bread in Fortaleza city (Ceará, Brazil).

#### 2.7.2. Antimicrobial Activity

In order to investigate the potential effects of Cry8Ka5 and Cry1Ac proteins on common gastrointestinal microbiota of mammals or phylogenetically close to that, growth inhibitory activity of bacterial and fungal was performed in liquid medium.

The study of growth inhibitory activity of bacteria exposed to concentrations of Cry8Ka5 and Cry1Ac ranging from 30.6 to 1,000 *μ*g/mL protein was performed according to the protocol described by Hissa et al. [32]. In sterile conditions, to each well of the microtiter plate was added 100 *μ*L of nutrient broth containing bacterial cells at a concentration of 10^7^ CFU/mL, followed by 100 *μ*L of each sterilized sample (sterilized using a Millipore filter of pore size 0.22 *μ*M) and ovalbumin at a concentration of 2,000 *μ*g/mL (non-Cry control), 50 mM sodium phosphate buffer pH 7.0 (negative control), or 0.4% formol (positive control). Then, the microtiter plates were subjected to reading at a wavelength of 600 nm in a microplate reader, followed by new readings every 4 h, up to a total of 24 h. For each sample, a triplicate against each bacteria species was performed. From the sample dilutions proceeded to calculate the minimum inhibitory concentration (MIC).

The growth inhibition assay of* C. albicans, C. tropicalis, P. anomala,* and* S. cerevisiae* by Cry8Ka5 and Cry1Ac proteins, in concentrations ranging from 30.6 to 1,000 *μ*g/mL, was performed according to the methodology described by Ribeiro et al. [33], with some modifications.

In microtitration plates (sterile) were added to each well 100 *μ*L of BHI broth (pH adjusted to 5.0) with a concentration of yeast-like cells corresponding to a reading of 0.05 (OD_600_), followed by 100 *μ*L of each sample of Cry8Ka5 and Cry1Ac proteins (sterilized using a Millipore filter of pore size 0.22 *μ*M), ovalbumin (2000 *μ*g/mL), sodium phosphate buffer 50 mM pH 7.0 (negative control), or 0.4% formol (positive control). Then, the plates were subjected to the reading at a wavelength of 600 nm in a microplate reader, followed by further readings every 6 h until a total of 48 h. To each sample, it was performed one triplicate to each yeast species. From the sample dilutions proceeded to estimate the minimum inhibitory concentration (MIC).

## 3. Results

### 3.1. Recombinant Proteins Production and Characterization

**Figure 1:**
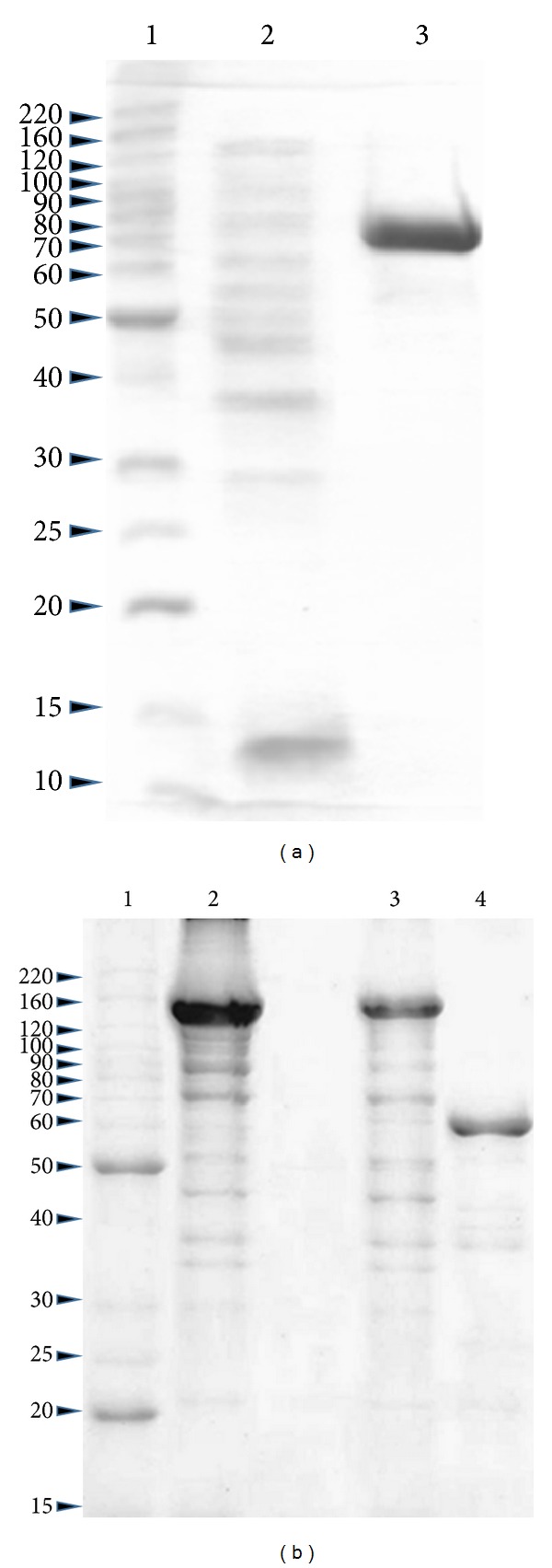
(a) Electrophoretic profile of the heterologous gene expression of Cry8Ka5 protein in polyacrylamide gel (12.5%) under reducing and denaturing conditions. Lane 1: molecular weight markers. Lane 2: peak not retained from the chromatography on the Ni-NTA resin. Lane 3: peak retained from the chromatography on the Ni-NTA resin eluted with 20 mM imidazole. (b) Electrophoretic profile of the heterologous gene expression of Cry1Ac protein in polyacrylamide gel (12.5%) under reducing and denaturing conditions. Lane 1: molecular weight marker; Lane 2: protoxin Cry1Ac not dialyzed; Lane 3: protoxin Cry1Ac dialyzed; Lane 4: toxin Cry1Ac after trypsinization and dialysis.

SDS-PAGE profiles of Cry8Ka5 and Cry1Ac recombinant proteins, as well as some steps of the expression and purification process, are shown in Figure 1. Both entomotoxins presented the expected molecular mass (near to 70.0 kDa). From the SDS-PAGE profiles it was possible to estimate the relative purity of each protein obtained using adequate software. Either for Cry8Ka5 or for Cry1Ac, the different batches of expression and purification presented relative purity which ranged from 75% up to 95%. Besides, Cry8Ka5 and Cry1Ac proteins showed to be active against* A. grandis* and* S. frugiperda *larvae, respectively, in the expected magnitude.

To confirm the identity of the proteins produced, they were electrotransferred from the electrophoresis gels to PVDF membranes and carried out for determination of N-terminal sequence. The N-terminal sequence obtained for Cry8Ka5 was SEGYDNKYFANPEVFAAPGGITTGIT, which is identical to the amino acid sequence deposited by the group that developed the mutant protein [6] with accession number G8XRZ1 at UniProtKB/Swiss-Prot database. Likewise, the N-terminal sequence obtained for Cry1Ac was IETGYTPIDISLSLTQFLLSEFVPGAGF, which is identical to multiple entries for the same protein (P05068, E3TBL1, Q6XLN7).

### 3.2. Cyto- and Genotoxicity of Cry8Ka5 and Cry1Ac Proteins

Table 1 shows the results of the evaluation of cytotoxicity in human peripheral lymphocytes of Cry8Ka5 and Cry1Ac proteins through MTT assay after 72 h of exposure. This classic assay for assessment of nonspecific cytotoxic effects of natural and synthetic compounds showed that, even at the highest concentration tested (1,000 *μ*g/mL), the toxins caused no significant effect on the viability of the lymphocytes, resulting in IC_50_ > 1,000 *μ*g/mL.

**Figure 2:**
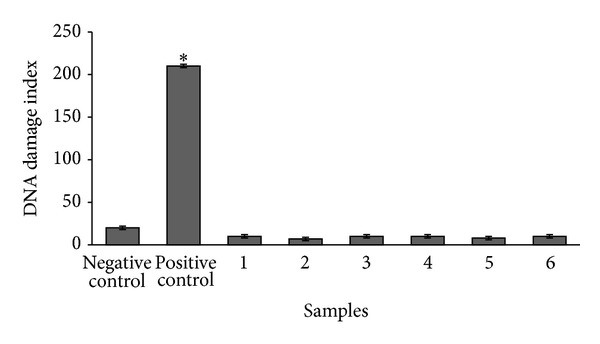
DNA damage index in human lymphocytes exposed for 24 h to Cry8Ka5 and Cry1Ac proteins. The 20 mM sodium phosphate buffer, pH 7.0, was used as negative control and doxorubicin at the concentration of 0.6 mM was used as positive control. 1, 2, and 3 correspond to Cry1Ac at concentrations of 100, 500, and 1,000 *μ*g/mL, respectively. 4, 5, and 6 correspond to Cry8Ka5 at concentrations of 100, 500, and 1,000 *μ*g/mL, respectively. All data correspond to means ± standard deviation of four independent experiments. *P < 0.05; compared to negative control by ANOVA/Newman-Keuls.

As for the evaluation of genotoxicity, the proteins did not cause relevant cellular DNA damage, since the outcomes were comparable to the result of the negative control, the 20 mM sodium phosphate buffer, pH 7.0 (Figure 2). The positive control, doxorubicin 0.6 *μ*M, produced DNA damage index higher than 200.

### 3.3. Hemolytic Activity

In Table 2, you can see the results of the hemolytic activity analysis of 2% human (types A, B, AB, and O), rabbit, and mice erythrocyte suspensions treated with Cry8Ka5 and Cry1Ac. The Cry8Ka5 protein, just like bovine serum albumin, did not show significant hemolytic activity (<1%) for all erythrocytes suspensions used. In contrast, the Cry1Ac toxin showed some hemolytic activity in all tested erythrocyte suspensions, which oscillated from 4.6% in rabbit to 16.6% in type AB human erythrocytes suspension. Given the low percentage of hemolysis detected, all samples tested showed IC_50_ > 1,000 *μ*g/mL.

### 3.4. AFM

**Figure 3:**
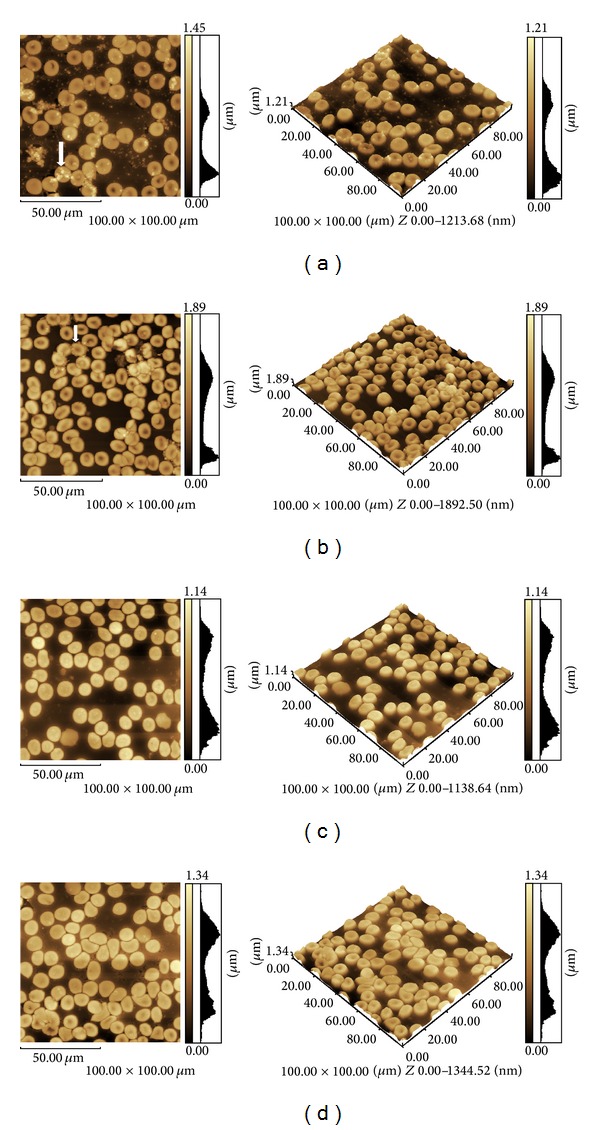
Human erythrocytes (type O) 2D and 3D images (100 × 100 *μ*m) captured by atomic force microscopy. (a) Blood film treated with 1,000 *μ*g/mL of Cry8Ka5 protein. (b) Blood film treated with 1,000 *μ*g/mL of Cry1Ac protein. (c) Blood film treated with 1,000 *μ*g/mL of bovine serum albumin. (d) Blood film untreated. Arrows indicate the visible presence of Cry proteins on the surface of the erythrocytes.

In order to contribute with further information to the hemolytic activity test, the cell membrane topography of human erythrocytes (type O, in whole blood after treatment for 1 h, at 37°C) was assessed with Cry8Ka5, Cry1Ac, and bovine serum albumin proteins at a concentration of 1,000 *μ*g/mL (Table 3). Seven parameters were analyzed in total. Almost all parameters of erythrocytes treated with Cry8Ka5 differed (*P* < 0.05) from those of the other treatments, Cry1Ac, albumin, and erythrocytes untreated. The only exception was the average *Z* parameter that was equal to that of albumin treated erythrocytes. The average *Z* obtained the average value data from the *Z* pixels that form a particle (an erythrocyte). The remaining samples, Cry1Ac and bovine serum albumin, caused significant differences (*P* < 0.05) in erythrocytes only in the volume parameter when compared to untreated erythrocytes. In Figure 3, there are illustrations in 2D and 3D of the erythrocytes treated with the proteins and control and with no treatment. In (A) and (B) you can view the Cry8Ka5 and Cry1Ac proteins, respectively, on the surface of erythrocytes or dispersed in the extracellular fluid. In addition, no visible abnormality or deformity can be found in erythrocytes treated with the proteins or when compared to those which were not exposed to any treatment.

### 3.5. Effects on* Artemia* sp

To contribute with further information to the cytotoxicity assessment, it was performed an alternative evaluation that uses* Artemia* nauplii. Cytotoxicity was estimated based on the occurrence of nauplii death. Then,* Artemia* nauplii 48 h old were exposed to Cry8Ka5, Cry1Ac, and bovine serum albumin proteins in various concentrations and also to the artificial seawater control for 24 h.

In Table 4, it is possible to observe that the Cry8Ka5 protein showed LC_50_ of 755.11 ± 86.88 *μ*g/mL, unique among the samples with LC_50_ lower than 1,000 *μ*g/mL (highest concentration).

### 3.6. Effects on Microorganisms

**Figure 4:**
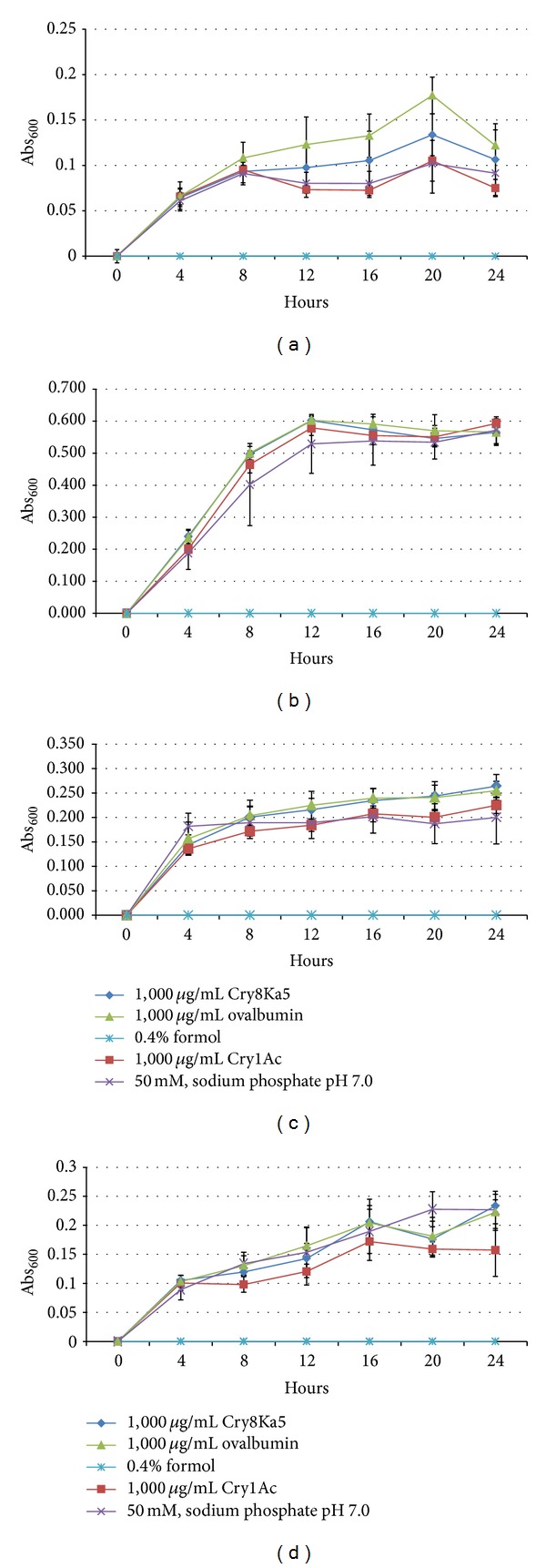
Growth curves of Gram-positive strains, (a)* Bacillus subtilis* and (b)* Staphylococcus aureus*, and Gram-negative bacteria, (c)* Enterobacter aerogenes* and (d)* Salmonella choleraesuis*, grown in nutrient broth containing the Cry8Ka5, Cry1Ac, and ovalbumin, all at a concentration of 1,000 *μ*g/mL, and non-protein controls, sodium 50 mM phosphate buffer, pH 7.0, and 0.4% formol. The coefficients of variation for each point were ≤10%. For all readings Abs_600_, Cry8Ka5, and Cry1Ac showed no significant difference (*P* > 0.05, one-way ANOVA) compared to the controls, ovalbumin, or 50 mM sodium phosphate buffer, pH 7.0.

**Figure 5:**
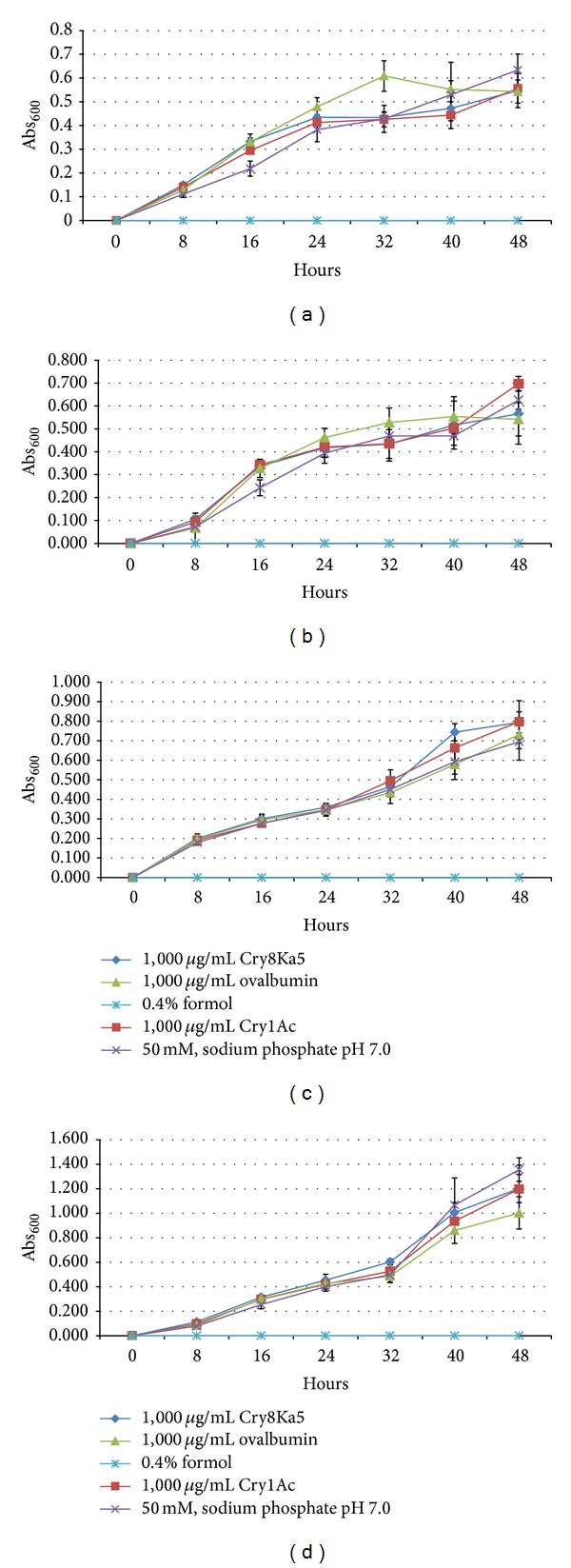
Growth curves of the yeast (a)* Candida albicans*, (b)* C. tropicalis*, (c)* Pichia anomala,* and (d)* Saccharomyces cerevisiae* grown in BHI broth, pH 5.0, containing Cry8Ka5, Cry1Ac, and ovalbumin proteins, all in concentration of 1,000 *μ*g/mL, and nonprotein controls, 0.05 M sodium phosphate buffer, pH 7.0, and 0.4% formol. The coefficients of variation for each point were ≤10%. For all readings Abs_600_, Cry8Ka5, and Cry1Ac showed no significant difference (*P* > 0.05, ANOVA) compared to the controls, ovalbumin, or 50 mM sodium phosphate buffer, pH 7.0.

Four bacterial strains, two Gram-positive (*B. subtilis* and* S. aureus*) and two Gram-negative (*E. aerogenes* and* S. choleraesuis*) and also four yeasts (*C. albicans, C. tropicalis, P. anomala,* and* Saccharomyces cerevisiae*) were exposed for 24 and 48 h, respectively, at concentrations ranging from 30.6 to 1,000 *μ*g/mL of Cry8Ka5, Cry1Ac, ovalbumin (negative protein control), 50 mM sodium phosphate buffer pH 7.0 (negative control), and 0.4% formol (positive control), to evaluate the effects of proteins on some microorganisms that comprise the microbiota of mammals or of those phylogenetically close. In Figure 4, it is presented the growth curves separately of each bacteria strains in the presence of the protein samples at the highest concentration tested (1,000 *μ*g/mL). For each kind of bacteria, it is possible to observe a linearity of growth and a clear overlay (*P* > 0.05 for each reading at OD_600_) for each sample tested, except for the treatment with formol that resulted in no growth. Similarly, in Figure 5, it is shown separately the growth curves of each kind of yeast when exposed to the protein samples. It can be seen that the growth curve obeys linearity and an evident overlay between the curves (*P* > 0.05 for each reading at OD_600_), except in the treatment with formol in which no growth was observed at all. Consequently, the MIC of the protein samples was > 1,000 *μ*g/mL, in both bacteria and yeasts tested.

## 4. Discussion

Even though the current GMO safety assessments seem to be efficient, several studies have pointed out failures or gaps in these procedures regarding noncoverage of important issues such as the evaluation of effects on cells of nontarget organisms (e.g., mammalian cells) and on the microbiota of the GMO consumer organism [15–18]. In this context, this study aimed to evaluate the Cry8Ka5 mutant protein and the Cry1Ac protein on their cyto- and genotoxic effects upon human lymphocytes, the presence of damage in erythrocytes of three mammalian species, cytotoxicity to* Artemia* sp., and antimicrobial effects.

Tests to assess the cytotoxic and genotoxic effects in peripheral lymphocytes of healthy humans have been extensively used for safety evaluation of natural and synthetic compounds present in high concentrations in foods and in the environment as contaminants or for the evaluation of new specific molecules with anticancer potential [27, 34]. Even at the highest concentration tested (1,000 *μ*g/mL), the Cry1Ac and Cry8Ka5 did not show any cytotoxic effects on lymphocytes. Likewise, these proteins at a concentration of 1,000 *μ*g/mL did not cause any lymphocyte DNA damage, showing no genotoxic effects according to the method used. Shimada et al. [35] and Bondzio et al. [18] also did not detect cytotoxic effects of the toxin Cry1Ab in bovine hepatocytes culture and epithelial cells culture from ovine rumen, respectively. Other Cry toxins, such as Cry4a and Cry11A did not show cytotoxic effects against human breast carcinoma line MCF-7 [36].

Studies addressing the effects of Cry proteins on the genetic material of healthy cells are extremely rare. However, Grisolia et al. [37] reported genotoxic effects in zebrafish larvae and embryos (a model for this type of study) in the presence of moderate doses (25–150 *μ*g/mL) of Cry1Aa, Cry1Ab, Cry1Ac, and Cry2A proteins separately or in combinations. The absence of cytotoxic and genotoxic effects of the Cry proteins tested on human lymphocytes can be reasonably explained by the lack of receptors for effective binding of these proteins to the surface of this kind of cells [38]. It is also worth noting that the evaluation of cyto- and genotoxicity in human lymphocytes with Cry proteins performed in this study is the first example of the use of these tests for the safety assessment of* Bt* toxins.

The effect of Cry8Ka5 and Cry1Ac toxins on the integrity of the erythrocytes membrane of rabbit, rat, and humans (types A, B, AB, and O) was also investigated. The reason for this is that the erythrocytes are the most abundant cells in various species of animals of the phylum chordata and are subject to various cytotoxic molecules, including proteins, called hemolysins. Thus, two tests were performed, the classic hemolytic activity in erythrocyte suspension (2%) and the erythrocyte cell membrane topography analysis in whole human blood (type O). In the evaluation of hemolytic activity, all suspensions of erythrocytes from different origins did not show hemolysis percentage higher than 1% when exposed to the highest concentration of Cry8Ka5 (1,000 *μ*g/mL). On the other hand, Cry1Ac protein showed percentage values of hemolytic activity at the highest concentration (1,000 *μ*g/mL) towards different erythrocyte samples, which ranged from 4.6 to 16.6%. However, according to the criteria of Bernheimer [29], results lower than 20% must be considered irrelevant given that certain animal species have inherent propensity of erythrocyte hemolysis.

Although the hemolytic activity test provides valuable results in the context of a safety assessment of a recombinant protein, not all harmful substances to cells cause severe cell membrane injury that culminate in disruption of the cell membrane and release of hemoglobin. Often, the damage to the membrane may be due to the low concentration of potentially cytotoxic molecule or on account of low affinity for membrane receptors. Such circumstances may lead to a slight loss of cell content or swelling due to slow influx of external fluid, in both cases, causing deformities in the cell. Therefore, the cellular membrane topography by AFM analysis can contribute with information about the integrity of the erythrocyte membrane exposed to Cry proteins. In the case of the exposure of human erythrocytes (type O) to Cry8Ka5 protein compared to native erythrocytes (untreated), differences were observed in the seven parameters. Even though these differences were significant from a statistical standpoint, the changes promoted by Cry8Ka5 were not too pronounced, and the average values of the parameters of this treatment were only 0.1 to 0.24 times larger or smaller than the values of the group not treated. The magnitude of this difference is not easy to explain, but it certainly was not able to promote hemolysis of the cells, even at a concentration where it is unlikely that a nontarget cell would be exposed. Another point worth mentioning is that all samples differed from the untreated group in the volume parameter which is perhaps the most important parameter because it provides clear signs of a possible influx of fluid into the cell given the hypotonic condition of the medium. Depending on the period of exposure to the treatment, this could lead to cell lysis. The possibility of using cell membrane topography analysis via AFM was initially raised by Brand et al. [39], who showed marked changes in the erythrocyte membrane parameters caused by treatment with dermaseptins, which are cytotoxic peptides isolated from the skin of the frog* Phyllomedusa hypochondrialis*. Another interesting fact is that it is possible to see clearly from the images in 2D and 3D the accumulation of Cry proteins, especially Cry8Ka5, on the erythrocytes surface. If there was any interaction of Cry proteins with membrane receptors, it was not able to unleash their classic effects of pore formation in the same magnitude that occurs in the midgut cells of target insects. In general, the proteins tested did not cause severe damage to erythrocytes, neither with hemolysis nor with pronounced alterations in the membrane.

Still in the context of the cytotoxicity assessing of Cry8Ka5 and Cry1Ac proteins, the survival rate of* Artemia* sp. nauplii was evaluated. The genus* Artemia* (Crustacea, Branchiopoda, and Anostraca), especially the species* Artemia salina* and* A. franciscana*, is used in a wide range of toxicological tests and research [40–42]. Since it is a low cost, fast, and very informative test and does not bring up ethical issues (because it does not inflict suffering to vertebrates), it was employed in this study. The Cry1Ac protein showed very high LC_50_ (> 1,000 *μ*g/mL), while the Cry8Ka5 showed lower value (755.11 *μ*g/mL). However, if we consider the LC_50_ of Cry8Ka5 to its main target, the cotton boll weevil (2.83 *μ*g/mL, according to Oliveira et al. [6]), we would conclude that the value found against* Artemia* is almost 270 times greater, which only reinforces the specificity of this protein. Moreover, hardly a nontarget organism would be exposed to Cry8Ka5 in such high concentration. Therefore, the results of the cytotoxicity assay with* Artemia* sp. corroborate the results of other tests performed with Cry8Ka5 but showed greater sensitivity to potential toxic effects of this molecule. This suggests the need of further testing with a larger number of different Cry molecules to confirm the potential of this microcrustacean as a biomarker for evaluating new* Bt* proteins.

One of the main questions about the effectiveness of the current methods for the safety assessment of recombinant proteins is the absence of tests that evaluate the impact of these molecules on the microbiota of mammals' gastrointestinal tract [18]. Also, there is a concern about the effects of these proteins on soil microorganisms, given the importance of maintaining the balance of ecosystems [43, 44]. In this context, we selected four bacteria and four yeasts, all common to human and animal microbiota, to assess the antimicrobial effects of the experimental proteins. Both Cry1Ac and Cry8Ka5 proteins showed no growth inhibition in liquid medium neither to the Gram-positive and Gram-negative bacteria nor to yeasts at a concentration of 1,000 *μ*g/mL. In fact, most reports of antibacterial activity of* Bt* proteins are related to the classes of Cry proteins with mosquitocidal activity, that is, Cry proteins with activity against Diptera [15, 16], which is not the case of the Cry proteins under study. Nevertheless, for some Cry toxins active against other classes of insects (such as Cry1Ab, Cry1D, and Cry3Aa), there are reports of antimicrobial effects against aerobic and anaerobic bacteria, with MICs ranging from 45 to 150 *μ*g/mL and with mechanisms of action related to the composition of the cell wall [17]. Hence, the absence of antibacterial activity of the Cry toxins tested may be related to cell wall composition of the strains used. As to the absence of antifungal activity of the Cry proteins, the composition of the fungal cell wall formed mainly of chitin and nonspecific binding sites for these proteins may explain the negative results obtained against fungi. Indeed, the absence of cytotoxic and genotoxic effects in mammalian cells and the negative effects on the microorganisms' growth are extremely positive characteristics in the context of Cry8Ka5 and Cry1Ac proteins safety assessment.

## 5. Conclusions

In conclusion, the Cry8Ka5 and Cry1Ac proteins showed no significant cytotoxicity or genotoxicity, even when tested at high doses under different methodologies, and have not shown antimicrobial activity against the tested microorganisms. Moreover, the methods employed in this study contributed with valuable information on the effects of Cry8Ka5 and Cry1Ac proteins on nontarget organisms, enhancing their potential for safe biotechnological applications. In addition, these assays could be also used to gather safety information on other Cry proteins beyond those obtained in the current safety assessment approach.

## Figures and Tables

**Table 1:** Cytotoxic activity of Cry8Ka5 and Cry1Ac proteins on human peripheral lymphocytes evaluated by MTT assay [3-(4,5-dimethylthiazol-2-yl)-2,5-difeniltetrazol] after 72 h exposure.

Proteins	IC_50_^a^ for human lymphocytes (*μ*g/mL)
Cry8Ka5	>1,000
Cry1Ac	>1,000

^a^Concentration able to inhibit 50% of human lymphocytes growth.

**Table 2:** Presence of hemolytic activity in different mammals' species erythrocytes suspensions at 2% treated with Cry8Ka5, Cry1Ac, and other protein and nonprotein controls.

Erythrocytes (2%)	Distilled water (positive control)		NaCl 0.9% (Negative control)		Cry8Ka5 1,000 *μ*g/mL			Cry1Ac 1,000 *μ*g/mL			Bovine serum albumin 1,000 *μ*g/mL		
	Abs_540_^a^	% Hem^b^	Abs_540_	% Hem	Abs_540_	% Hem	IC_50_^c^ *μ*g/mL	Abs_540_	% Hem	IC_50_ *μ*g/mL	Abs_540_	% Hem	IC_50_ *μ*g/mL
Human blood type													
A	0.456	100	0.119	0	0.115	0	>1,000	0.173	11.8	>1,000	0.092	0	>1,000
B	0.675	100	0.107	0	0.110	0.7	>1,000	0.140	4.9	>1,000	0.089	0	>1,000
AB	0.648	100	0.112	0	0.106	0	>1,000	0.220	16.6	>1,000	0.089	0	>1,000
O	0.932	100	0.106	0	0.113	0.75	>1,000	0.150	4.7	>1,000	0.087	0	>1,000
Rabbit	0.681	100	0.109	0	0.107	0	>1,000	0.141	4.6	>1,000	0.096	0	>1,000
Rat	0.446	100	0.143	0	0.126	0	>1,000	0.205	13.9	>1.000	0.126	0	>1,000

The absorbance values (Abs) and % hemolysis are averages of triplicates. The standard deviations were lower than 20%. ^a^Wavelength (nm) used to measure the release of hemoglobin; ^b^% Hemolysis = (Abs_540_ sample − Abs_540_ of the negative control) × 100/Abs_540_ of the positive control; ^c^Concentration capable of causing 50% hemolysis of the cells.

**Table 3:** One-dimensional, two-dimensional, and three-dimensional parameters of erythrocytes morphology treated with Cry8Ka5, Cry1Ac, or bovine serum albumin at a concentration of 1,000 *μ*g/mL measured by atomic force microscopy.

Parameters	Cry8Ka5	Cry1Ac	Bovine Serum albumin	Untreated^g^
Standard width (*μ*m)	9.71 ± 1.17^abc^	8.62 ± 0.92	8.44 ± 0.94	8.48 ± 0.82
Perimeter (*μ*m)	33.15 ± 3.59^abc^	28.68 ± 1.86	28.45 ± 2.08	29.42 ± 2.20
Average *Z* (*μ*m)	0.76 ± 0.08^ac^	1.18 ± 0.14^de^	0.78 ± 0.07^f^	1.00 ± 0.12
Area excluding voids (*μ*m^2^)	72.97 ± 12.15^abc^	58.48 ± 7.66	58.36 ± 7.95	61.08 ± 7.92
Area including voids (*μ*m^2^)	73.74 ± 11.45^abc^	58.43 ± 7.66	58.38 ± 7.66	61.16 ± 7.84
Surface area (*μ*m^2^)	74.96 ± 12.58^abc^	61.83 ± 8.12	59.36 ± 8.01	62.24 ± 8.04
Volume (*μ*m^3^)	55.94 ± 10.72^abc^	69.56 ± 14.46^de^	45.66 ± 7.04^f^	61.45 ± 10.70

Values are means ± standard deviations of 100 measures; ^a^ *P* < 0.05 (one-way ANOVA) for Cry8Ka5 compared to Cry1Ac; ^b^ *P* < 0.05 (one-way ANOVA) for Cry8Ka5 compared to bovine serum albumin; ^c^ *P* < 0.05 (one-way ANOVA) for Cry8Ka5 compared to untreated; ^d^ *P* < 0.05 (one-way ANOVA) for Cry1Ac compared to bovine serum albumin; ^e^ *P* < 0.05 (one-way ANOVA) for Cry1Ac compared to untreated; ^f^ *P* < 0.05 (one-way ANOVA) to bovine serum albumin compared to untreated.

^g^Erythrocytes not exposed to treatment.

**Table 4:** Cytotoxicity evaluation of Cry8Ka5, Cry1Ac, and protein and nonprotein controls against *Artemia* sp. nauplii after 24 h exposure.

Samples	Concentrations (*μ*g/mL)	Mortality^a^ (%)	LC_50_^b^ (*μ*g/mL)
Cry8Ka5	1,000	66.67	755.11 ± 86.88 (623.93–978.60)^c^
	500	26.67	
	250	6.67	
	125	0	

Cry1Ac	1,000	10	>1,000

Bovine serum albumin	1,000	3.33	>1,000

Artificial seawater	—	0	—

Temephos^d^	—	—	0.16

^a^Values are means of triplicate for each sample. The standard deviations were less than 5% of means; ^b^Sample concentration capable of killing 50% of *Artemia* sp. nauplii (confidence limit of 95%); ^c^(lower limit, upper limit); ^d^Taken from Souza et al. [26].

## References

[1] Prieto-Samsonov DL, Vázquez-Padrón RI, Ayra-Pardo C, González-Cabrera J, de la Riva GA (1997). *Bacillus thuringiensis*: from biodiversity to biotechnology. *Journal of Industrial Microbiology and Biotechnology*.

[2] Mendelsohn J, Baselga J (2003). Status of epidermal growth factor receptor antagonists in the biology and treatment of cancer. *Journal of Clinical Oncology*.

[3] Romeis J, Meissle M, Bigler F (2006). Transgenic crops expressing *Bacillus* thuringiensis toxins and biological control. *Nature Biotechnology*.

[4] Gatehouse AMR, Ferry N, Edwards MG, Bell HA (2011). Insect-resistant biotech crops and their impacts on beneficial arthropods. *Philosophical Transactions of the Royal Society B: Biological Sciences*.

[5] Sanahuja G, Banakar R, Twyman RM, Capell T, Christou P (2011). *Bacillus thuringiensis*: a century of research, development and commercial applications. *Plant Biotechnology Journal*.

[6] Oliveira GR, Silva MC, Lucena WA (2011). Improving Cry8Ka toxin activity towards the cotton boll weevil (*Anthonomus grandis*). *BMC Biotechnology*.

[7] Delaney B, Astwood JD, Cunny H (2008). Evaluation of protein safety in the context of agricultural biotechnology. *Food and Chemical Toxicology*.

[8] O'Callaghan M, Glare TR, Burgess EPJ, Malone LA (2005). Effects of plants genetically modified for insect resistance on nontarget organisms. *Annual Review of Entomology*.

[9] Yu H, Li Y, Wu K (2011). Risk assessment and ecological effects of transgenic *Bacillus thuringiensis* crops on non-target organisms. *Journal of Integrative Plant Biology*.

[10] Noteborn HPJM, Bienenmann-Ploum ME, van den Berg JHJ, Takeoko GR, Teranishi R, Engel KH (1996). Safety assessment of the *Bacillus thuringiensis* insecticidal crystal protein CRYIA(b) expressed in tomato. *Genetically Modified Foods: Safety Issues*.

[11] Gill M, Ellar D (2002). Transgenic *Drosophila* reveals a functional *in vivo* receptor for the *Bacillus thuringiensis* toxin Cry1Ac1. *Insect Molecular Biology*.

[12] Broderick NA, Raffa KF, Handelsman J (2006). Midgut bacteria required for *Bacillus thuringiensis* insecticidal activity. *Proceedings of the National Academy of Sciences of the United States of America*.

[13] Kim H-S, Yamashita S, Akao T (2000). *In vitro* cytotoxicity of non-Cyt inclusion proteins of a *Bacillus thuringiensis* isolate against human cells, including cancer cells. *Journal of Applied Microbiology*.

[14] Mesnage R, Clair E, Gress S, Then C, Székács A, Séralini G-E (2013). Cytotoxicity on human cells of Cry1Ab and Cry1Ac Bt insecticidal toxins alone or with a glyphosate-based herbicide. *Journal of Applied Toxicology*.

[15] Yudina TG, Konukhova AV, Revina LP, Kostina LI, Zalunin IA, Chestukhina GG (2003). Antibacterial activity of Cry- and Cyt-proteins from Bacillus thuringiensis ssp. israelensis. *Canadian Journal of Microbiology*.

[16] Revina LP, Kostina LI, Dronina MA (2005). Novel antibacterial proteins from entomocidal crystals of *Bacillus thuringiensis* ssp. *israelensis*. *Canadian Journal of Microbiology*.

[17] Yudina TG, Brioukhanov AL, Zalunin IA (2007). Antimicrobial activity of different proteins and their fragments from *Bacillus thuringiensis* parasporal crystals against clostridia and archaea. *Anaerobe*.

[18] Bondzio A, Stumpff F, Schön J, Martens H, Einspanier R (2008). Impact of *Bacillus thuringiensis* toxin Cry1Ab on rumen epithelial cells (REC)—a new *in vitro* model for safety assessment of recombinant food compounds. *Food and Chemical Toxicology*.

[19] Bondzio A, Lodemann U, Weise C, Einspanier R (2013). Cry1Ab treatment has no effects on viability of cultured porcine intestinal cells, but triggers Hsp70 expression. *PLoS ONE*.

[20] Bradford MM (1976). A rapid and sensitive method for the quantitation of microgram quantities of protein utilizing the principle of protein dye binding. *Analytical Biochemistry*.

[21] Laemmli UK (1970). Cleavage of structural proteins during the assembly of the head of bacteriophage T4. *Nature*.

[22] Grossi-de-Sa MF, de Magalhães MQ, Silva MS (2007). Susceptibility of *Anthonomus grandis* (cotton boll weevil) and *Spodoptera frugiperda* (fall armyworm) to a Cry1Ia-type toxin from a Brazilian *Bacillus thuringiensis* strain. *Journal of Biochemistry and Molecular Biology*.

[23] Edman P (1964). Determination of amino acid sequences in protein. *Thrombosis et Diathesis Haemorrhagica*.

[24] Burim RV, Canalle R, Lopes JLC, Vichnewski W, Takahashi CS (2001). Genotoxic action of the sesquiterpene lactone centratherin on mammalian cells in vitro and in vivo. *Teratogenesis, Carcinogenesis, and Mutagenesis*.

[25] Mosmann T (1983). Rapid colorimetric assay for cellular growth and survival: application to proliferation and cytotoxicity assays. *Journal of Immunological Methods*.

[26] Souza TM, Farias DF, Soares BM (2011). Toxicity of Brazilian plant seed extracts to two strains of *Aedes aegypti* (Diptera: Culicidae) and nontarget animals. *Journal of Medical Entomology*.

[27] Lima PDL, Leite DS, Vasconcellos MC (2007). Genotoxic effects of aluminum chloride in cultured human lymphocytes treated in different phases of cell cycle. *Food and Chemical Toxicology*.

[28] Merker MP, Levine L (1986). A protein from the marine mollusc *Aplysia californica* that is hemolytic and stimulates arachidonic acid metabolism in cultured mammalian cells. *Toxicon*.

[29] Bernheimer AW (1988). Assay of hemolytic toxins. *Methods in Enzymology*.

[30] Costa-Lotufo LV, Khan MT, Ather A (2005). Studies of the anticancer potential of plants used in Bangladeshi folk medicine. *Journal of Ethnopharmacology*.

[31] Finney DJ (1971). *Probit Analysis*.

[32] Hissa DC, Vasconcelos IM, Carvalho AFU (2008). Novel surfactant proteins are involved in the structure and stability of foam nests from the frog *Leptodactylus vastus*. *Journal of Experimental Biology*.

[33] Ribeiro SFF, Agizzioa AP, Machado OLT (2007). A new peptide of melon seeds which shows sequence homology with vicilin: partial characterization and antifungal activity. *Scientia Horticulturae*.

[34] Militão GCG, Dantas INF, Ferreira PMP (2012). *In vitro* and *in vivo* anticancer properties of cucurbitacin isolated from *Cayaponia racemosa*. *Pharmaceutical Biology*.

[35] Shimada N, Kim YS, Miyamoto K, Yoshioka M, Murata H (2003). Effects of *Bacillus thuringiensis* Cry1Ab toxin on mammalian cells. *Journal of Veterinary Medical Science*.

[36] Corrêa RFT, Ardisson-Araújo DMP, Monnerat RG, Ribeiro BM (2012). Cytotoxicity analysis of three *Bacillus thuringiensis* subsp. *israelensisδ*-endotoxins towards insect and mammalian cells. *PLoS ONE*.

[37] Grisolia CK, Oliveira R, Domingues I, Oliveira-Filho EC, Monerat RG, Soares AMVM (2009). Genotoxic evaluation of different *δ*-endotoxins from *Bacillus thuringiensis* on zebrafish adults and development in early life stages. *Mutation Research*.

[38] Bravo A, Gill SS, Soberón M (2007). Mode of action of *Bacillus thuringiensis* Cry and Cyt toxins and their potential for insect control. *Toxicon*.

[39] Brand GD, Leite JRSA, Silva LP (2002). Dermaseptins from *Phyllomedusa oreades* and *Phyllomedusa distincta*: anti-*Trypanosoma cruzi* activity without cytotoxicity to mammalian cells. *The Journal of Biological Chemistry*.

[40] Acey RA, Bailey S, Healy P, Jo C, Unger TF, Hudson RA (2002). A butyrylcholinesterase in the early development of the brine shrimp (*Artemia salina*) larvae: a target for phthalate ester embryotoxicity?. *Biochemical and Biophysical Research Communications*.

[41] Favilla M, Macchia L, Gallo A, Altomare C (2006). Toxicity assessment of metabolites of fungal biocontrol agents using two different (*Artemia salina* and *Daphnia magna*) invertebrate bioassays. *Food and Chemical Toxicology*.

[42] Mohamed ZA (2007). First report of toxic *Cylindrospermopsis raciborskii* and *Raphidiopsis mediterranea* (Cyanoprokaryota) in Egyptian fresh waters. *FEMS Microbiology Ecology*.

[43] Prihoda KR, Coats JR (2008). Aquatic fate and effects of Bacillus thuringiensis Cry3Bb1 protein: toward risk assessment. *Environmental Toxicology and Chemistry*.

[44] Prihoda KR, Coats JR (2008). Fate of *Bacillus thuringiensis* (*Bt*) Cry3Bb1 protein in a soil microcosm. *Chemosphere*.

